# Determinants of perinatal mortality in public secondary health facilities, Abuja Municipal Area Council, Federal Capital Territory, Abuja, Nigeria

**DOI:** 10.11604/pamj.2020.37.114.17108

**Published:** 2020-10-02

**Authors:** Ugochukwu Uzoechina Nwokoro, Tukur Dahiru, Abdulhakeem Olorukooba, Clement Koelengoen Daam, Hyelshini Samuel Waziri, Ayo Adebowale, Ndadilnasiya Endie Waziri, Patrick Nguku

**Affiliations:** 1 Nigeria Field Epidemiology and Laboratory Training Program, Abuja, Nigeria,; 2 Department of Community Medicine, Faculty of Medicine, Ahmadu Bello University, Zaria, Nigeria,; 3 Department of Epidemiology and Medical Statistics, Faculty of Public Health, University of Ibadan, Ibadan, Nigeria

**Keywords:** Perinatal death, antenatal care, secondary health facility, Nigeria

## Abstract

**Introduction:**

in Nigeria, perinatal mortality rate remains high among births at the health facility. Births occur majorly at the secondary healthcare level in Abuja Municipal Area Council (AMAC) of the Federal Capital Territory (FCT). Identifying factors influencing perinatal deaths in this setting would inform interventions on perinatal deaths reduction. We assessed perinatal mortality and its determinants in public secondary health facilities in AMAC.

**Methods:**

delivery and neonatal data from two selected public secondary health facilities between 2013 and 2016 were reviewed and we extracted maternal socio-demographics, obstetrics and neonatal data from hospital delivery, newborns´ admissions and discharge registers. Data were analyzed using descriptive statistics and Cox proportional hazard models (α = 5%).

**Results:**

perinatal mortality rate was 129.5 per 1000 births. Asphyxia 475 (34.0%), neonatal infection 279 (20.0%) and prematurity 242 (17.3%) accounted for majority of the 1,398 perinatal deaths. Unbooked status [aHR = 1.8 (95% CI 1.4 - 2.2)], antepartum haemorrhage [aHR = 2.8 (95% CI 1.2 - 6.7)], previous perinatal death [aHR = 2.3 (95% CI 1.7 - 3.1)] and maternal age ≥ 35 years [aHR= 1.4 (95% CI 1.0 - 1.8)] were associated with increased risk of perinatal death.

**Conclusion:**

perinatal mortality in the studied hospitals was high. Determinants of perinatal death were unbooked antenatal care (ANC) status, antepartum haemorrhage, previous perinatal death and high maternal age. Reducing perinatal deaths would require improving antenatal care attendance with healthcare staff identifying and targeting women at risk of pregnancy complications.

## Introduction

Perinatal mortality (PM) is the death of a fetus between 28 weeks of gestation and the first week after delivery. It is one of the indicators often used for assessing maternal and child health in any nation. Globally, it is estimated that about 7.5 million babies die annually during the perinatal period [1] and 98.0% of these deaths occur in developing countries. In sub-Saharan Africa, while under-5 mortality rate has declined over the last two decades, the pace of reduction in neonatal mortality has been much slower mainly due to huge contributions of perinatal deaths. New born deaths or babies that die in the first 28 days of life still account for a quarter of child deaths in the region [2]. In Nigeria, deaths among new born babies before they reach their first (69 per 1,000 births) and fifth (128 per 1,000 births) birthday remain a problem of public health importance [3]. The 2013 Nigeria demographic health survey noted declines of 26 percent and 31 percent in infant and under-5 mortality respectively in the preceding 15 years, however, perinatal mortality rate (PMR) increased from 39 per 1000 births in 2008 to 41 per 1000 births in 2013 representing approximately five percent increase over the five year period. The PMR in Nigeria is unacceptably high ranging from 60 to 130 per 1000 births among health facility births [4-10]. The recent PMR trend in Nigeria if unchecked is likely to pose a serious challenge to the attainment of the sustainable development goal on health (SDG 3) which aims at ensuring healthy lives for all and ending preventable deaths of newborns and under-five year old children by the year 2030.

Despite the high PMR in Nigeria and the FCT in particular, few studies have been conducted to assess perinatal mortality and its determinants at the secondary healthcare level. Nearly all the health facility-based studies previously conducted on perinatal mortality in the FCT were done in tertiary health facilities. These studies having excluded perinatal deaths in secondary health facilities which offer specialist comprehensive and newborn care as is obtained in tertiary health facilities might have under estimated the perinatal mortality indices in the area council and the FCT. Public secondary health facilities in Abuja Municipal Area Council of the FCT not only offer antenatal and delivery services to women with seemingly low-risk pregnancies but are also referral centers for high-risk or complicated obstetric and newborn cases in the area council. Understanding the determinants of perinatal deaths at this level of healthcare would help to inform the health care staff as well as policy makers on areas where clinical care and public health interventions could improve perinatal outcomes in the area council and other parts of the country. This study aimed to determine the perinatal mortality rate and factors associated with perinatal mortality among secondary health facility births in Abuja Municipal Area Council of the FCT.

## Methods

**Study area and setting:** the study was conducted in Abuja Municipal Area Council, one of the six area councils in the FCT, Abuja. Abuja is the capital city of Nigeria and is located in the geographical centre of the country. In 2016, the population of the FCT and Abuja Municipal Area Council was estimated to be 3,419,323 and 1,894,513 respectively. Women of child bearing age and pregnant women were projected to be 416,793 and 94,726 respectively in the area council. Abuja Municipal Area Council has six public secondary health facilities. This study was conducted in two public secondary health facilities namely: Asokoro District Hospital and Nyanya General Hospital randomly selected by balloting. These health facilities have specialist obstetrics and gynecology departments as well as paediatric departments with new born special care units. They offer 24 hour emergency obstetric and newborn special care. These centers each have an annual delivery of between 1200 and 1500 and they are usually the health facilities where most pregnant women resident in the area council present for comprehensive obstetric and newborn care. They also serve as referral centers for the many primary health care facilities located within and outside the area council.

**Study design:** this study involved a 4-year retrospective review of records in the selected public secondary health facilities covering the period from January 1^st^, 2013 to December 31^st^, 2016.

**Study population:** the study population was babies delivered after 28 weeks gestation in public secondary health facilities in Abuja Municipal Area Council of the FCT and their mothers.

### Eligibility criteria

**Inclusion criteria:** all babies delivered after 28 weeks of gestation between 1^st^ January, 2013 and 31^st^ December, 2016, in the selected health facilities as well as their mothers were included in the study. Babies admitted within the first seven days of birth at the newborn special care units of the selected health facilities over the study period were also included in the study.

**Study Instruments:** a structured data collection form consisting of the following sections: maternal socio-demographic data, obstetric history, prenatal interventions/treatment, intrapartum findings, fetal and perinatal outcome was used for data collection.

**Data collection procedure:** data from the mothers´ delivery registers as well as the babies´ admission and discharge registers at the newborn special care units of the selected health facilities were extracted and entered into the structured data collection form. Data extracted included maternal age, antenatal booking status, parity, educational status, employment status, previous obstetric history, antenatal antepartum conditions, intrapartum complications, gestational age at delivery, birth weight, first- and fifth-minute Apgar scores, fetal sex, newborn special care unit admission, perinatal complications and probable causes of perinatal deaths.

**Data analysis:** data were coded and statistical analysis conducted using Microsoft Excel and Epi info version 7.1.5.2 software. Frequencies and proportions were computed as descriptive statistics. The perinatal mortality rate, stillbirth rate and early neonatal death rate were equally computed. A modified version of the Wigglesworth classification of causes of perinatal mortality was used to classify the probable causes of perinatal mortality [11]. At the level of bivariate, the association between perinatal mortality and explanatory variables was determined using Cox proportional hazard model (α=5.0%). Variables that were found to be significant at the level of bivariate were included in the multivariate analysis in order to identify the determinants of perinatal mortality. The indicator of the status variable are the cases of perinatal mortality. The time to event variable is the life span of the fetus which covers the period of 28 weeks of gestation and the first week after delivery. Any dead fetus within this interval will attract a code 0 and 1 if otherwise. However, fetus where information on the survival status between 28 weeks of gestation and the first week after delivery could not be ascertained is said to be censored and the Cox proportional hazard model factors this in during iteration.

**Ethical considerations:** ethical approval for the study was sought and obtained from the Health Research Ethics Committee of the Health and Human Services Secretariat of the Federal Capital Territory Administration (FCTA), Abuja (FHREC/2016/01/64/26-08-16). Permission to access hospital data was sought and obtained from the managements of the selected secondary health facilities. Consent was not obtained from the mothers because there was no direct individual contact with patients; however, all data were de-identified before entry into the data collection form to ensure confidentiality.

## Results

There were a total of 10,797 deliveries over the four year period covered by the study. Majority of the mothers 10,269 (95.1%) had some level of formal education, 9,718 (90.0%) were married, 9,287 (86.0%) were aged 35 years or younger and 8,935 (82.8%) booked for antenatal care (Table 1). Of the 10,797 deliveries, 5,673 (52.5%) were males, 6,702 (62.1%) were delivered at term and 9,349 (88.5%) were of normal birth weight (Table 2). The overall perinatal mortality rate was 129.5 per 1000 births with stillbirth rate and early neonatal death rate of 29.5 per 1000 births and 103.1 per 1000 live births respectively (Table 3). Overall, asphyxia related conditions were the most common probable cause of perinatal mortality over the study period. These accounted for 475 (34.0%) of all 1,398 perinatal deaths. Other probable causes of perinatal mortality were early neonatal infections accounting for 279 (20.0%), immaturity 242 (17.3%), macerated stillbirths without congenital malformations 149 (10.7%), lethal congenital malformations 4 (0.3%) and others 249 (17.8%). Table 4 shows the crude and adjusted hazard ratios for factors influencing perinatal death. Marital status, mothers´ place of residence, type of gestation, Apgar score ≤ 6, birth weight, ANC booking status, previous history of perinatal death, antepartum heamorrhage, obstructed labour, high parity, maternal HIV infection, mode of delivery and fetal sex were associated with increased risk of perinatal death, however, on multivariate analysis, maternal age, marital status, place of residence, ANC booking status, multiple gestation, birth weight, antepartum haemorrhage and previous history of perinatal death remained significantly associated with increased risk of perinatal mortality (Table 4).

**Table 1:** socio-demographic and antenatal characteristics of mothers who were delivered of their babies in selected secondary health facilities, Abuja Municipal Area Council, FCT, Abuja, 2013 - 2016

Variable	Frequency (n=10,797)	%
**Age (years)**		
<20	201	1.9
20 - 34	9,086	84.2
≥35	1,510	13.9
**Marital status**		
Single	1,050	9.7
Married	9,718	90.0
Widowed	29	0.3
**Educational status**		
No formal education	528	4.9
Primary	924	8.6
Secondary	4,981	46.1
Tertiary	4,364	40.4
**Employment status**		
Employed	6,914	64.0
Unemployed	3,883	36.0
**Mother’s place of residence**		
Within Abuja Municipal Area Council	9,066	84.0
Outside Abuja Municipal Area Council	1,731	16.0
**Antenatal care booking status**		
Booked	8,935	82.8
Unbooked	1,862	17.2
**Parity**		
Primiparous	4,827	44.7
Multiparous	5,445	50.4
Grand multiparous	525	4.9
**Antepartum conditions**		
Pregnancy induced hypertension	64	0.6
Pre-eclamptic toxaemia	14	0.1
Eclamptic toxaemia	2	0.02
Previous caesarian section scar	369	3.4
VDRL positive	340	3.1
HIV positive	614	5.7
Previous perinatal death	114	1.1

**Table 2:** characteristics of births at the selected secondary health facilities, Abuja Municipal Area Council, Federal Capital Territory, Abuja, 2013 - 2016

Variable	Frequency (n=10797)	%
**Fetal sex**		
Male	5,673	52.5
Female	5,134	47.5
**Gestational age at delivery**		
Preterm	2,124	19.7
Term	6,702	62.1
Post term	1,971	18.3
**Mode of delivery**		
Spontaneous vaginal delivery	8,703	80.6
Assisted vaginal delivery (Forceps/vacuum delivery)	89	0.8
Elective caesarian section	415	3.8
Emergency caesarian section	1,590	14.7
**Type of gestation**		
Single	10,563	97.8
Twin	232	2.1
Higher-order	2	0.02
**Birth weight**		
Extreme low birth weight	33	0.3
Very low birth weight	47	0.4
Low birth weight	622	5.8
Normal birth weight	9,349	88.5
Macrosomia	505	4.7

**Table 3:** stillbirth rates, early neonatal death rates and perinatal mortality rates, selected secondary health facilities, Abuja Municipal Area Council, Abuja, 2013 - 2016

Variable	Frequency
Total number of births	10,797
Number of stillbirths	318
Stillbirth rate (per 1000 births)	29.5
Total number of live births	10,479
Number of early neonatal deaths	1080
Early neonatal death rate (per 1000 live births)	103.1
Total number of perinatal deaths	1,398
Perinatal mortality rate (per 1000 births)	129.5

**Table 4:** multivariate Cox proportional hazards regression model showing adjusted hazard ratios for the risk factors for perinatal mortality in secondary health facilities, Abuja Municipal Area Council, Federal Capital Territory, Abuja, 2013 - 2016

Covariates	Crude hazard ratio (95% CI)	Adjusted hazard ratio (95% CI)
**Marital status**		
Single	33.0 (25.3 - 42.8)^**^	11.9 (8.7 - 16.5)^**^
Married		
**Place of residence**		
Outside AMAC	1.4 (1.3 - 1.5)^**^	14.7 (10.6 - 20.4)^**^
Within AMAC		
**Type of gestation**		
Twin or higher-order	5.4 (3.1 - 9.6)^*^	9.8 (2.1 - 11.3)^**^
Singleton		
**APGAR score at 5 minutes**		
>6	0.7 (0.6 - 0.8)^**^	0.0005 (0.0001 - 0.0037)^**^
≤6		
**Birth weight**		
Low birth weight and Macrosomia	2.8 (2.7 - 3.0)^**^	8.7 (6.6 - 11.4)^**^
Normal birth weight		
**ANC booking status**		
Unbooked	3.9 (3.1 - 4.9)^*^	1.8 (1.4 - 2.2)^**^
Booked		
**Maternal age**		
≥35	1.0 (1.0 - 1.1)	1.4 (1.0 - 1.8)^*^
<35		
**Previous history of perinatal death**		
Yes	46.5 (36.4 - 59.5)^*^	2.3 (1.7 - 3.1)^**^
No		
**Ruptured uterus**		
Yes	23.4 (8.7 - 62.8)^*^	2.0 (0.6 - 6.6)
No		
**Antepartum haemorrhage**		
Yes	18.2 (8.1 - 40.8)^*^	2.8 (1.2 - 6.7)^*^
No		
**Obstructed labour**		
Yes	3.8 (1.4 - 10.2)^*^	0.6 (0.2 - 1.7)
No		
**Prolonged pregnancy**		
Yes	3.0 (1.6 - 5.7)^*^	1.0 (0.5 - 1.9)
No		
**Parity**		
≥5	1.1 (1.0 - 2.3)^*^	1.2 (0.8 - 1.8)
<5		
**HIV status**		
Positive	1.5 (1.0 - 2.3)^*^	1.2 (0.8 - 1.8)
Negative		
**Mode of delivery**		
Operative delivery	1.1 (1.0 - 1.1)^*^	1.0 (0.7 - 1.3)
Vaginal delivery		
**Fetal sex**		
Male	1.3 (1.0 - 1.6)^*^	1.1 (0.9 - 1.5)
Female		

^*^Significant at p-value <0.05; ^**^significant at p-value <0.001

## Discussion

This study found a high perinatal mortality rate of 129.5 per 1000 births. The high perinatal mortality rate observed in this study has important implications because perinatal mortality is regarded as a key indicator of the maternal and child health status of any population as well as a major contributor to overall under-five mortality. Also hospital-based data potentially underestimate the true rates of perinatal mortality because newborns are not followed up after discharge from the hospital and only 36% of births in Nigeria are delivered in health facilities [3] and out of hospital deliveries are likely to be associated with poorer outcomes. Secondary health facilities in Abuja Municipal Area Council offer specialist, comprehensive as well as emergency obstetrics and newborn care and therefore are referral centers for complicated obstetric and newborn cases from other health facilities within and outside the area council. This might have accounted for the high perinatal mortality rate observed in this study. The high perinatal death rate found in this study contrasts with the low rates reported from developed countries [2, 12]. However, it is similar to reports from several facility-based studies in Nigeria which have reported high rates [4-8, 10, 13, 14].

Asphyxia, early neonatal infection and immaturity were the main probable causes of perinatal mortality in this study. The results from our study are similar to reports from several studies which have reported these as the leading cause of perinatal deaths [6, 10, 15, 16]. The increased deaths due to asphyxia may be due to the late referral of intra-uterine hypoxia cases to these hospitals and also possibly due to delayed or poor early neonatal resuscitation. Globally, the main direct causes of newborn deaths are reported to be preterm birth, severe infections and asphyxia [17, 18] with prematurity playing a prominent role in developing countries [19]. Maternal parity of five and above, obstructed labor, postdatism and ruptured uterus were found to be risk factors for perinatal death at bivariate analysis; however, these were not significant at multivariate analysis. However, being unmarried was found to be associated with increased risk of perinatal mortality compared to being married. This might have been because married women are more likely to have partner support leading to increased social and economic support and are more likely to afford antenatal and intrapartum care services at the health facilities. Also women´s status in the society have been noted to interact with other factors, albeit in unclear terms to influence perinatal death [2]. Other studies conducted in the United States and Zimbabwe found unmarried marital status to be an independent predictor of perinatal death [20, 21].

Mothers aged 35 years and older had an increased risk of perinatal mortality compared to those aged below 35 years. This finding corroborates the findings of the study by Fawole *et al*. in Nigeria and another study on low and middle income countries [10, 22] as well as the findings of systematic reviews on the association between perinatal mortality and advanced maternal age in high income countries [23-25]. Women 35 years and older have been reported to be more likely to experience a term stillbirth than women aged below 35 years with stillbirths in this group of women reported to be more likely due to major congenital anomalies, maternal disorders, mechanical causes or associated obstetric factors compared to women younger than 35 years [24]. Place of mother´s residence was found to be a determinant of perinatal mortality in this study. Babies born to mothers who lived outside Abuja Municipal Area Council had an increased risk of perinatal death compared to those born to mothers who lived within the area council. The finding of a higher risk of perinatal mortality among this category of women could be as a result of having to commute longer distances to assess antenatal care and intrapartum care services at the health facilities compared to those who lived within the area council thus making accessibility of antenatal and delivery services more difficult. The findings of this study corroborate the findings of Akello *et al*. who found an increased risk of perinatal death among babies whose mothers travelled greater distances to access care [26].

Some studies in Nigeria [8, 10, 15] and elsewhere [27, 28] have reported unbooked status and lack of prenatal care to increase the risk of perinatal death. We found that newborns of mothers who were unbooked for antenatal care had about twice the risk of perinatal death compared to newborns of mothers who booked for antenatal care. This is because antepartum complications in women who are unbooked for antenatal care services are less likely to be detected on time and thus resulting in poor perinatal outcomes in this group of women. Antepartum haemorrhage was associated with an increased risk of perinatal mortality in this study. This finding is similar to findings from other studies in Nigeria which have reported antepartum haemorrhage and experiencing a pregnancy complication as important risk factors for perinatal mortality especially where late presentation and delay in instituting appropriate interventions result in poor perinatal outcomes [4, 8, 14, 29]. Babies born as twins or higher-order births were found to have increased risk of dying in the perinatal period compared to singletons. Multiple pregnancies are high risk pregnancies and have been found to significantly increase the risk of perinatal mortality compared to singleton pregnancies because of increased risk of complications from prematurity, low birth weight and intrauterine growth restriction. Studies in Nigeria [4, 15, 30] and India [28] found multiple pregnancies to increase the risk of perinatal death. Dahiru also reported an increased risk of first day mortality and early neonatal death among multiple births in Nigeria [31].

We also found newborns with low birth weight and macrosomia to have significantly increased risk of perinatal death compared to normal birth weight newborns. Low birth weight and fetal macrosomia have been reported to be significant determinants of perinatal death. Although previous studies in Abuja Municipal Area Council have found no significant association between birth weight and perinatal mortality [8, 10], other studies have found strong associations between low birth weight and perinatal death in Nigeria [6, 15] as well as in other countries [28, 32, 33]. Also, fetal macrosomia has been reported to be associated with increased risk of intrapartum stillbirths [34, 35]. As documented by Fawole *et al*. unbooked antenatal care status with the attendant lack of prenatal care as well as poor intrapartum and neonatal care services may have statistical interactions in the contributions of not only low birth weight but also fetal macrosomia to perinatal mortality in our setting [10]. An Apgar score of at least seven at five minutes after birth was found to be protective against perinatal death. The high risk of perinatal death associated with birth asphyxia suggests poor state of early neonatal care services in our setting. This finding is similar to the findings of other studies in Nigeria which have reported birth asphyxia and low Apgar scores at five minutes to be independent predictors of perinatal death [6-10, 15, 36]. Basic neonatal resuscitation has been reported to have a great impact in reducing intrapartum-related neonatal deaths in sub-Saharan Africa [37].

**Limitations:** this being a hospital-based study, its findings may not reflect outcome of deliveries outside health facilities. Also findings of this study may not capture perinatal outcomes in private hospital deliveries considering that the study was conducted in public secondary health facilities.

## Conclusion

The perinatal mortality rate in public secondary health facilities in Abuja Municipal Area Council of the FCT was high. Newborn deaths were mainly due to birth asphyxia, neonatal infection and prematurity. Un-booked antenatal care status, previous history of perinatal death, antepartum haemorrhage, high maternal age, low birth weight and macrosomia as well as multiple pregnancies were associated with increased risk of perinatal mortality. Improving ANC attendance and delivery care services at the secondary health facility level with healthcare staff identifying and targeting women at risk of obstetric complications and ensuring appropriate neonatal resuscitation at birth may improve perinatal outcomes.

### What is known about this topic

- Perinatal mortality is a major contributor to neonatal and under-five mortality;
- Perinatal mortality is highest in sub-Saharan Africa and Asia;
- Majority of perinatal deaths are intra-partum related.

### What this study adds

- The perinatal mortality rate among secondary health facility births in Abuja Municipal Area Council is high as a result of high early neonatal death rate;
- Neonatal infection in the early neonatal period is a major contributor to perinatal mortality;
- The important role of identifying and targeting women at risk of pregnancy complications by healthcare staff could play in reducing perinatal deaths.
